# The Medical Use of Music

**Published:** 1883-04

**Authors:** George L. Beardsley

**Affiliations:** Birmingham, Conn.


### Popular Science Department.

### THE MEDICAL USES OF MUSIC.

The spells of music have always been themes for speculation. The earliest inquiries concerned this loan of Apollo to man. Melody was no sooner revered than its birth was deciphered, and among the primitive calculations was the mission of song. Its winsome appeal to passion ; its faculty of lulling heart-aches, scattering the vapors of a burdened spleen, or smoothing the rugged pathway of grievous duty ; its aid to genius ; its friendship to humor ; its inspiration to holy offerings, these varied values of melody touched the harp-strings of the early student and made him sing of music as of some far off welking descended, the offspring of the rustling river-reeds, or the whispers from well-disposed Olympus.

Its startling energy in animating, and curious ascendancy over, morbid experiences suggested its utility long ago as a cure for pain, equally as a method of worship or an overture for assistance.

Pythagoras, who referred the origin of melody to the gambols of the spheres, prescribed sonnets to those laboring under aberration of mind whether sensible or feigned. His trials of it were confined to those who were fretted by delusions. Sacred writ instances the power of sweet strains to break vials of wrath, as in the case of Saul incensed against David, who exhibited a species of mania, or madness fed by jealousy, which condition is, according to the present reading of the mind diseased, a variety of true derangement. It is affirmed of the wise Thales that under his auspices an imminent plague was banished from Sparta by the skillful exercise of musical instruments. The probability of such an antidotal element in minstrelsy lacks warrant in the light of the current views on the genesis of disease, but the notice of the story sustains the statement that the ancients expected a virtue in music to operate against the disorders of nature. Capella who flourished in the fifth century and wrote a quaint compound of allegory and the liberal sciences, relates the prescription of music to cure fevers and tells of its positive success. Hearing is reported by Asclepiades to have been restored by stimulating the organism with frequent blowing of a trumpet. The same practice of playing the lyre and singing lays before mad patients (apparently first suggested by Pythagoras) was continued by Xenocrates, who figured among the Greeks in 396 B. C. The confidence in the serviceableness of music in disease does not appear to have waned as successive experiments measured its utility—the indications for the remedy rather increased, for in the writings of the popular Greek schoolmaster, Theophrastus, cases are detailed of the cure, by music, of rabies and the shock or depression induced by the bites of vipers or venomous serpents. Not all these reports are to be received as the sportive wiles of some deft juggler—they are far from the impossible. The terror of wounds inflicted by rabid animals and that black despair so proverbial a symptom among those thus bitten are remediless save by exorcising or some sort of machination of electro-biology that shall charm away the bug-bear. Reason's discourses to a hydrophobic patient are not sovereign, and the use of sounds in silvery cadence may be a remedy yet to be appraised as prolific of comfort as the amulet, or the royal touch.

In the second century Aulus Gellius, a Roman grammarian, and author of the "Attic Nights," described a case of sciatica cured by harmonics. In the latter medical literature the employment of incantations to ease pain and pacify the furies is often noted, and the measure is recommended with emphasis not repulsive. In the time of Luther the people were profoundly impressed with the special antagonism of mellow airs to the wiles of maleficent beings. This zealous propagandist of a new confession defined music as a "bitter enemy of Satan." The persuasion that mellifluous sonatas are not congenial to the devil, and that insanity in all its varieties, for ages expounded as the curse of an angry god, can be appeased by the richest euphony, is not discredited, nor lightly esteemed among those who determine law to disease. The use of music now preferred in insane asylums is an evidence that we are possessed of a clearer ken of the maniac's disturbance. This is an era in which the sentinels of science are gaining ground surely—when heathen frauds are being buried at wisdom's gate, and ere long it may be affirmed of the pioneers in the healing of the mind diseased, that in the "golden tongue" they have found a sway over grim despair.

The same data which made sensible the service of modulations with the insane will explain the propriety of doctoring any of the other disturbances of the nervous system, particularly the functional, with minstrel performances. The notion that mirth is bracing and that the hornpipe mocks a funeral, is no raw increment to reason's capital. The ancients had a saw that the three best physicians were "*mens laeta*, requies, dieta moderata." Macbeth when he "throws physic to the dogs," forgot to ask for pipes and whistles. The charms of music are as far-famed as Eden's serpent. In sickness its persuasiveness seems irrepressible and full many a time when decay is long spun out and death defers his call to fill full the cup of gnawing weariness, harmony comes as pain's seducer. To those suffering ideal ills, or who can call up any ache at leisure, the dyspeptic, the man with clogged liver or big spleen, the brigade of victims not of youthful indiscretions, but inmates of painted sepulchres and patrons of white-robed medicasters, to all these pilgrims of Despond some Terpischorean strains, the soul-stirring symphonies of a Mozart, the rollicking choruses of a darkey troupe, the thrilling anthem, the melting ditty will dispense the nepenthe that no drug depot ever inventoried. The story told of Farinelli curing Philip of Spain of suicidal intentions induced by ill-health, is a valuable confirmation of the possibility of controlling nervous depression by sonatas. Philip had passed into a state of profound despondency, continuing thus for days, secluded, not even willing to have an attendant to shave him. The Queen, confident that music would unyoke the bond of cold melancholy, ordered a concert to be given in a room near the King's chamber. The ravishing warbles of Farinelli at once overcame the King, who ordered the singer into his audience. Farinelli continued the soft impeachment until the King consented to resume his duties, was shaved and appeared in public. To make the cure genuine the Queen required Farinelli to sing daily for some time. No return of the "blues" to Philip, King of Spain, was ever afterward noticed. The affection, mal de pays or nostalgia, is said to be greatly amenable to minstrel ditties—the banjo may yet be found a specific for home-sickness. With the Romans female musicians or psaltriæ were hired to play before the love-lorn ; the Swiss were fired from withering despair to courage dreadless by the Rans des Vaches ; the pibroch made the Scot wax hot to die a hero. It is not the courage of wild fiction to conclude that the South Germans rarely resort to suicide because of music's grip on madness, and more than one to-day whose griefs of digestion or cultivated cough have made a rover after Siloam's waters, would forget all twinges if once moved by the electric trills of a Jenny Lind, a Gottschalk, or a Paginini. The gospel of sweet rhyme has not yet been proclaimed of all its affluence. The rewards of song will not be ripe, nor can its coronation be confirmed until the energy hid away in music's sanctuary is brought out, and made to combat every pain that does violence to temperate reason or nice content.—*By George L. Beardsley, of Birmingham, Conn., in New England Medical Monthly.*

